# Desiderata for delivering NLP to accelerate healthcare AI advancement and a Mayo Clinic NLP-as-a-service implementation

**DOI:** 10.1038/s41746-019-0208-8

**Published:** 2019-12-17

**Authors:** Andrew Wen, Sunyang Fu, Sungrim Moon, Mohamed El Wazir, Andrew Rosenbaum, Vinod C. Kaggal, Sijia Liu, Sunghwan Sohn, Hongfang Liu, Jungwei Fan

**Affiliations:** 1 Division of Digital Health Sciences, Department of Health Sciences Research, Mayo Clinic, Rochester, MN USA; 2 Department of Cardiovascular Medicine, Mayo Clinic, Rochester, MN USA; 3 Advanced Analytics Service Unit, Department of Information Technology, Mayo Clinic, Rochester, MN USA

**Keywords:** Medical research, Health care

## Abstract

Data is foundational to high-quality artificial intelligence (AI). Given that a substantial amount of clinically relevant information is embedded in unstructured data, natural language processing (NLP) plays an essential role in extracting valuable information that can benefit decision making, administration reporting, and research. Here, we share several desiderata pertaining to development and usage of NLP systems, derived from two decades of experience implementing clinical NLP at the Mayo Clinic, to inform the healthcare AI community. Using a framework, we developed as an example implementation, the desiderata emphasize the importance of a user-friendly platform, efficient collection of domain expert inputs, seamless integration with clinical data, and a highly scalable computing infrastructure.

## Introduction—natural language processing in digital medicine

The furor surrounding artificial intelligence (AI) in healthcare has led to rapid advancement in digital medicine across multiple clinical specialties, including intensive care,^1^ cardiovascular medicine,^2^ neurology,^3^ oncology,^4^ and ophthalmology^5^; primarily enabled by big data generated through the digitization of healthcare. As a majority of clinical information in digitized clinical data is embedded within clinical narratives, unlocking such information computationally through natural language processing (NLP) is of paramount value to advancing healthcare AI.^6^

NLP approaches can generally be divided into either symbolic or statistical techniques. A recent review^7^ has shown that symbolic techniques predominate in clinical NLP, one major reason being that dictionary or rule-based methodologies suffice to meet the information needs of many clinical applications,^8,9^ whereas statistical NLP requires labor-intensive production of a set of labeled examples. Another consideration is the low error tolerance for clinical use cases. Tuning accuracy in symbolic systems is transparent and tractable via resource updates (e.g., terms or filters). This advantage is particularly applicable to clinical use cases, where the targets to extract are well-defined within a self-contained application, and authoring interpretable rules reduces the labor in massive data annotation especially where expert time is restricted. Unlike symbolic methods, conclusively fixing errors in statistical systems is difficult without incorporating symbolic techniques, such as post-processing rules. For instance, with symbolic NLP, detecting the state code “CA” erroneously as cancer is relatively straightforward to fix by adding contextual rules (e.g., look ahead for zip code or look behind for city name). Fixing this problem in statistical NLP systems would include time-consuming productions of training annotations, feature engineering, and retraining—all with no guarantee of successful resolution.

For end-to-end healthcare AI applications, NLP primarily serves as a method for information extraction rather than as a full-fledged standalone solution,^10,11^ i.e., NLP output is typically taken as part of a larger input set, or used to systematically extract training target values, for downstream AI models.

Despite its prevalence in clinical use cases, symbolic NLP lacks portability^12^ due to variations in documentation practices across clinicians. It follows that if the NLP component is not portable, then any AI that relies on it for feature extraction will also face similar issues. It is therefore desirable to address these issues, as they present a significant barrier to healthcare AI development and adoption.

## Desiderata for the implementation of an NLP development and delivery platform

Here, we present several recommendations for developing NLP toolsets that were derived from difficulties encountered, while developing clinical NLP at the Mayo Clinic.

### Desideratum I: To innovate, domain expertise must be collected and preserved

In many of our translational projects, we observed that the primary bottleneck in NLP development for healthcare AI was the high time and resource cost. Specifically, domain knowledge is necessary to successfully navigate clinical narratives, necessitating engagement of expensive clinical expertise. NLP definitions created from domain expertise should therefore be preserved and reused so as to reduce duplicate labor and accelerate innovation.

Central storage of this domain expertise has an additional benefit in that it allows for large-scale analysis. The limited portability of symbolic systems fundamentally stems from each clinical NLP project, presenting a single-perspective, dataset specific, definition of clinical concepts. We believe a crowd-sourcing approach can be used to resolve this issue. Having a multitude of expert perspectives for each concept allows downstream applications to learn from hundreds of different semantic views. Any target use case can then leverage the custom-weighted semantics of such views, bridging the portability gap across projects. To successfully implement this paradigm, the NLP definitions must be collected into a central location from which the embedded clinical domain knowledge can be harnessed across many projects.

### Desideratum II: To facilitate, toolsets should engage and empower domain experts

To collect domain expertise, experts must be incentivized to utilize the centralized platform. A common criticism of many publicly available NLP pipelines is that they are difficult to use and customize,^13^ particularly without NLP expertise. Clinical expertise is however held by clinicians, who do not typically have this expertise. For many of our projects, this disparity caused inefficiency by requiring a middleman to handle NLP development.^8,11,14–17^ To facilitate adoption and development, a clinical NLP platform should be easily customizable via a user-friendly front-end.

Given the known limitations of statistical methods, we observed that our collaborating clinicians and scientists have predominantly preferred rule-based NLP systems for integration into their analytics pipelines^8,11,14,15,18^ due to the relative ease of customization for improving information extraction performance: a preference that should optimally also be reflected in clinical NLP system implementations to incentivize usage. Here, we emphasize the NLP tasks that benefit most from directly interacting and soliciting clinical expert input, as is the case for concept extraction. For lower level linguistic tasks, such as tokenization and sentence chunking, statistical models can still be a decent option especially when adequate training data is available.

### Desideratum III: To accelerate, NLP platforms should be responsive and scalable

The amount of data needed to successfully develop healthcare AI can be a bottleneck, as we found that many of our projects involved datasets that would take months to process per iteration. Additional complications arise when these projects move out of development into clinical care, where near-instantaneous responsiveness is expected. In the general domain, this is handled by leveraging horizontal scaling and parallel computing, which, while beginning to be employed for clinical research and operations,^19–21^ remains largely inaccessible to casual end users.

To address challenges intrinsic to large data needs, clinical NLP infrastructure should (a) be developed with big data capabilities. Aside from operational throughput, scalability would naturally benefit any research, involving statistical power. Loading data into a big data platform and selecting only the desired data for processing are, however, challenging tasks.^22^ NLP solutions should (b) ideally integrate with existing EHR data stores on big data infrastructure to remove manual selection, retrieval, and loading of an impractically large set of documents prior to execution. In line with the prior desiderata, cohort definitions utilizing these integrations should be definable via an end user-friendly front-end.

## In pursuit of the desiderata—Mayo Clinic enterprise NLP platform to accelerate clinical NLP and crowd-source knowledge engineering

Here, we present NLP as a Service (NLPaaS), an example implementation of these desiderata as done at the Mayo Clinic.

An NLP task can be defined as an amalgamation of four components, which we will refer to throughout this section:

- Projects: Defines what to extract
- Cohorts: Defines what data to use
- Resources: User-customizable artifacts for project or cohort definitions
- Jobs: Executing a project on a cohort to produce NLP results

**Fig. 1:**
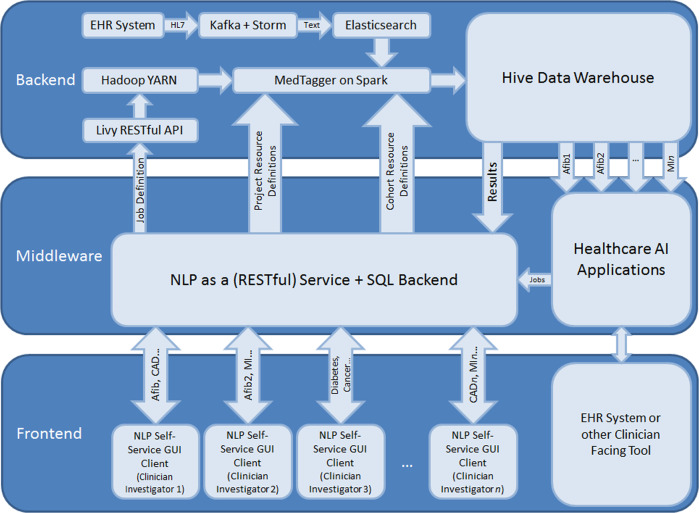
NLPaaS architecture diagram.

An overview of the platform is shown in Fig. 1, and is presented in detail in the ensuing sections.

### Backend—utilizing big data platforms to support high-throughput NLP (Desideratum III)

The backend performs the computation involved with an NLP task, while handling the large datasets involved in a distributed manner to enable responsive computation within reasonable timeframes.

To address scalability and responsiveness, the implementation presented here was built on top of the Hortonworks Data Platform,^23^ a distribution of the Hadoop ecosystem which provides a software framework for users to distribute computational (via a paradigm termed MapReduce) and storage (via the Hadoop File System) tasks across a cluster of computing nodes.

To support responsive document search and retrieval in a distributed environment, we used Elasticsearch (ES; an open-source distributed search engine) as the document source. The framework leveraged existing institutional infrastructure^17^ that produced an ES document store updated in near-real-time that contains all narratives and associated metadata generated in the clinical practice.

Storing these documents in ES in a standardized format with consistent metadata keys enabled high-throughput retrieval and simplified NLP pipeline integration. Using the ES-Hadoop library,^24^ documents corresponding to any arbitrary cohort definition can be retrieved to populate a Spark^25^ (an in-memory implementation of the MapReduce paradigm) dataset, which is then consumed by the NLP component.

The NLP component extends MedTagger,^26^ a rule-based NLP engine built on the UIMA framework.^27^ To support distributed execution, we extended this pipeline by encapsulating it as a Spark mapping operation, with a document collection as input, and the set of NLP annotations and metadata extracted from the documents as output. Pseudocode for the mapping function can be found in Supplementary Methods.

To enable customizability without requiring separate software packages for each job, MedTagger was modified to retrieve its project definition from middleware as opposed to using embedded or file-based resources.

The large volume of input data being processed in parallel results in output annotations of high velocity and volume. While traditional relational database management systems (RDBMS) will have difficulty handling this, it is still desirable to store these annotations in an SQL accessible format, as it is both computationally accessible and traditionally used for many data science pipelines to handle and analyze data sets.

The output is thus written to Apache Hive,^28^ an implementation of a data warehouse that can be queried using SQL in a distributed manner, and is therefore capable of handling the large volume of data being generated.

Job resource allocation and scheduling (i.e., load balancing across the cluster and determining which machines to use) is handled by Yet Another Resource Negotiator (YARN),^29^ while the actual job configuration and subsequent call to YARN is handled through the Livy REST API.

To run a job, the corresponding cohort definition is first retrieved and used to determine the number of documents involved, from which the amount of computational resources to allocate is derived. This information is then sent alongside the identifiers of the resources associated with the job to Livy to initiate NLP execution.

### Middleware—programmatic access to centralized resource and job management (Desideratum I)

Middleware, implemented using Spring Boot (https://spring.io/), supplies a centralized repository for NLP artifacts produced via clinical expertise and bridges users to the backend by providing a RESTful API for resource and job management, including creation, editing, and deletion. It also supplies the backend with these user-defined resources. A SQL database was used as storage for persistence of resource and job definitions.

Beyond resource and job management, middleware is ideal to handle auditing to comply with data protection standards, as it is the gateway through which data access occurs. All REST calls and associated metadata, including the date/time, user, and request content, were logged.

### Front-end—an user-friendly interface for self-service NLP functionality (Desideratum II)

Middleware helps facilitate computational access to NLP management and execution, but does little to improve the end user experience and to incentivize clinician participation and usage. As such, a graphical user interface (GUI) built using the Java Swing^30^ framework was implemented to encapsulate the functionality provided by middleware in a user-accessible manner.

Each project defines what to extract from text, as well as how to determine the semantic context of extracted items, e.g., whether it is negated, a historical mention, who the subject is, etc. The self-service layer also functions as a crowd-sourcing proxy that amasses diverse user perspectives and routes them to middleware’s knowledge repository.

**Fig. 2:**
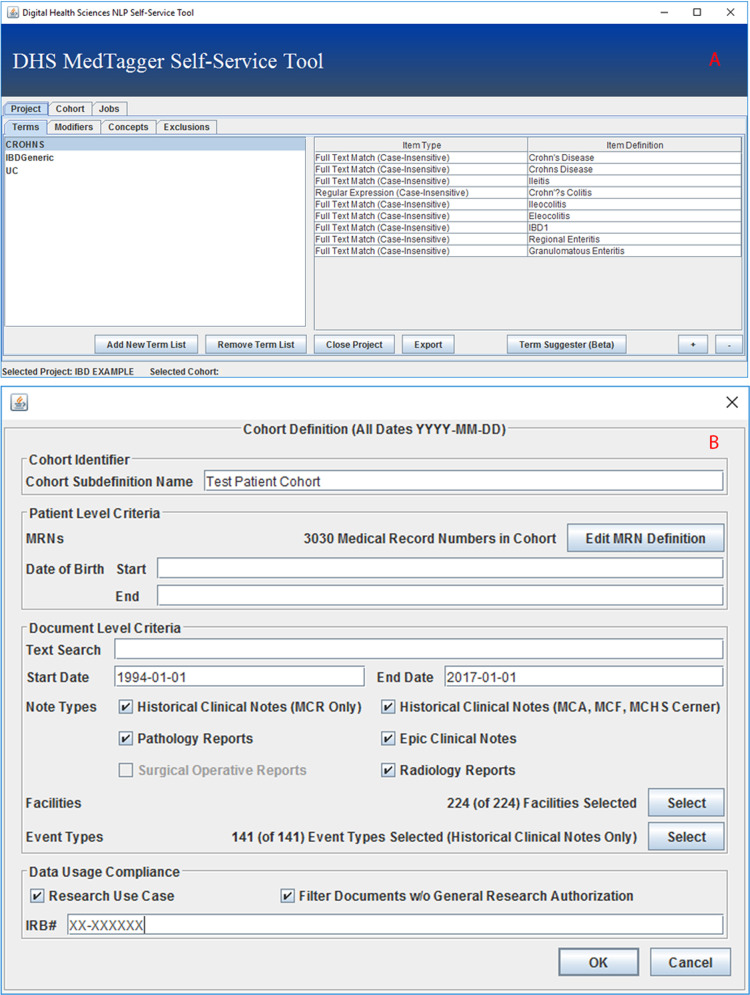
The NLPaaS clinical concept and cohort definition interfaces. Clinical concept definition interface **a** and cohort definition interface **b**.

The GUI lists the user’s projects, and allows projects to be defined and modified; allowing clinicians to create groups of textual patterns that together represent some normalized concept (Fig. 2a), and define the document types and sections that contain such concepts. It also allows users to fine-tune the ConText^31^ algorithm that is used by MedTagger for semantic context detection, although a ruleset that has been found to have a high performance on clinical narratives is loaded by default. The GUI also allows users to arbitrarily define the cohort to be used (Fig. 2b). Fields that can be defined include demographic information, such as the record numbers, start, and end dates, of a set of patients, as well as document level information, such as the document type, originating hospital service, or radiological exam modality and type.

Upon selection of both a project and a cohort, users are prompted to initiate a job. Middleware is triggered to schedule a job with the current project and cohort definitions. Job management (listing, progress, deletion, and retrieving results from jobs) is also made available in the GUI.

## Trialing NLPaaS at the Mayo Clinic

To test the NLPaaS platform, we solicited usage for 61 unique projects relating to clinical AI efforts at the Mayo Clinic with an average cohort size of 6.6 million documents from 01 May 2019 through 30 September 2019. There were 269 distinct clinical concepts defined in these projects.

Based on audit logs from middleware during the specified time period, an average of 256 executor threads were used for job execution (16 nodes × 16 cores), and on average a project required 3.9 h of cluster computation, equivalent to 247.9 h of continuous computation on a standard quad-core workstation. Please refer to Table 1 for details.

**Table 1:** NLPaaS pilot usage metrics from 01 May 2019 through 30 September 2019—cluster statistics and resulting workstation estimates are determined based on a calculated average of 256 executor threads (16 executor nodes × 16 cores).

Metric Name	Value
Number of projects	61.0
Number of jobs	246.0
Number of pilot users	13.0
Number of unique concepts (across all projects)	269.0
Average number of unique concepts per project	5.0
Average number of documents per job	6,624,651.1
Average number of jobs ran per project	4.0
Average job runtime (cluster)	1.0 h
Average project runtime (cluster; avg job runtime × avg number of jobs per project)	3.9 h
Average document throughput (cluster)	6,896,784.1 documents per hour
Total job runtime (cluster)	236.3 h (9.8 days)
Estimated equivalent average job runtime (quad-core workstation)	61.5 h (2.6 days)
Estimated equivalent average project runtime (quad-core workstation)	247.9 h (10.3 days)
Estimated equivalent total job runtime (quad-core workstation)	15,122.8 h (630.1 days)

Here, we highlight two projects from differing clinical settings that utilized NLPaaS.

### Identification of patients with cardiac sarcoidosis

Cardiac sarcoidosis is a rare disease where clumps of white blood cells form in heart tissue. Diagnosis is elusive and commonly made in a probable or presumptive manner based on clinical and imaging criteria, which must be assembled via chart review. Because of these difficulties and the accompanying inconsistency between different abstractors, computational automation of this process was desired.

As much of the required information is in unstructured text, NLPaaS was used by a cardiologist to identify relevant concepts, which led to the definition of six clinical concepts to identify patients with cardiac sarcoidosis.

Users expressed that NLPaaS was very intuitive to use, after they received a 15 min in-person tutorial and a 13-page manual for reference. Additionally, the analysis-friendly structured format of the output allowing for out-of-the-box filtering of the data according to the user’s needs was indicated to be a major advantage over other toolsets.

The feedback however also indicated that identification of exactly what constituted any given clinical concept required multiple iterations of trial-and-error, and that semiautonomous rule generation based on user input was desirable.

### Identification of silent brain infarction events

Silent brain infarctions (SBIs), brain lesions presumed to be due to vascular occlusion, are commonly detected as incidental findings in patients without clinical manifestations of stroke via neuroimaging.^32,33^ Despite serious consequences and high prevalence, identification of SBI events is challenging as no overt symptoms are presented and the screening required for detection is nonroutine, resulting in an absence of diagnoses in structured data.^32–34^

Descriptions of these events are frequently documented in radiology reports as text, rendering NLPaaS an ideal tool to assist in identification of SBI cases. Through iterative refinement conducted by a neurologist and neuroradiologist, 36 SBI-related terms were generated and grouped into three semantic concepts.^16^

User feedback indicated that NLPaaS was easily adoptable and usable due to being distributed as a standalone executable with easy-to-follow instructions. The integration of multiple data sources (e.g., neuroimaging reports and clinical notes) substantially reduced the effort of data collection and preprocessing.

## Discussion

It is important to note that the NLPaaS platform is only one of many possible implementations representing these desiderata. Indeed, a growing number of projects within the community independently manifest some of these desiderata: from end user accessibility,^35^ to high-throughput NLP,^36^ to knowledge collection and aggregation.^37^ These echoes corroborated that the summarized principles did not come out of vacuum. Additionally, there are additional factors, such as staffing, infrastructure, and budgeting to consider but were intentionally left outside our planned scope. While promoting convergence toward the desiderata, we should bear in mind that implementations will naturally vary between institutions due to existing workflows and infrastructure. Similarly, the presented performance metrics should only be used to gauge the potential gains from deploying big data technologies, but not to naively endorse the solution’s performance after following these desiderata.

To that end, we present the NLPaaS platform here as it was implemented at the Mayo Clinic for two reasons: (1) to demonstrate the benefits of adopting these desiderata, and (2) to provide an implementation reference.

From the pilot phase, we demonstrated that NLPaaS was found to be useful and sufficiently intuitive to attract clinician users, which is vital to successfully crowd-source knowledge. A set of 269 distinct clinical concepts covering a wide range of clinical specialties being produced from the 61 projects demonstrates the potential of such a centralized NLP platform to collect and crowd-source domain knowledge at a large scale.

Our success in engaging users from numerous projects within a five month timeframe is testament to the benefits of following these desiderata, and the number of unique clinical concepts collected in middleware during this pilot period suggested that our automated collection of domain knowledge to be working.

In addition, we demonstrated the utility of implementing NLPaaS on a big data platform, allowing for a greater number of tuning iterations and shorter cycles of NLP development. Such efficiency strongly incentivized usage, especially in situations with limited funding. Had the pilot projects been done on standard quad-core workstations, an average of 247.9 h of execution per project would have been needed, instead of a mere 3.9 h.

Despite these successes, a tradeoff was made for ease-of-use: several advanced NLP subtasks, such as dependency parsing, are not currently accessible. In the future, we plan to enable an advanced features portion of the GUI that leverages UIMA’s inherent modularity to customize these functions.

Formal evaluation of crowd sourcing for semantic portability was deferred, as the existing projects have not yet attained a sufficient number of converging concepts for a proper evaluation. Future work will also include a formal satisfaction survey to record detailed user feedback.

## Conclusion

Feature extraction via NLP is critical for successful healthcare AI. Despite this, current clinical NLP pipelines are difficult to use, scale, and customize. Additionally, because NLP tends to be a feature extraction method rather than standalone, there is a tendency towards symbolic systems that are easier to adopt and use, but lack portability.

In this paper, we have outlined several desiderata addressing these limitations for consideration when designing NLP platforms. We also presented the implementation, successes, and limitations of a platform built using these principles at the Mayo Clinic.

## Supplementary information

Supplementary Materials 1

## Data Availability

Middleware audit logs are not publicly available due to privacy and security concerns, and would be difficult to distribute to researchers not engaged in IRB-approved collaborations with the Mayo Clinic.

## Code availability

All software components as utilized in the example implementation of this platform are open-source projects: MedTagger can be found at https://github.com/OHNLP/MedTagger, the elastic stack can be found at (distributable binary): https://www.elastic.co/ or (source code): https://github.com/elastic. All other components can be found as top-level Apache projects.
