# Supplementary material for: Desiderata for delivering NLP to accelerate healthcare AI advancement and a Mayo Clinic NLP-as-a-service implementation
Source: NPJ Digit Med. 2019 Dec 17;2:130. doi: 10.1038/s41746-019-0208-8 (PMC6917754; doi:10.1038/s41746-019-0208-8)
Supplement: Supplementary file 1 — Supplementary Materials 1 [file 41746_2019_208_MOESM1_ESM.docx]

| *initializeMedTaggerPipeline()*  CAS uimaCas *// Central Data Structure for Analytics Tasks Supplied by UIMA*  Set[NLPAnnotation] results = new Set[NLPAnnotation]  **for** document **in** partition:  *loadDocumentIntoUimaCAS(*document,uimaCas*);*  *runMedTaggerPipeline(*uimaCas*)*  **for** result **in** *getResultsFromCAS(*uimaCas*):*  results.*add(convertResultToNLPAnnotation(*result*))*  *resetAndClearCAS(*uimaCas*)*  **return** results |
| --- |

Supp. Methods 1 - Pseudocode for the Spark Mapping Function
